# Divergence of climbing escape flight performance in *Morpho* butterflies living in different microhabitats

**DOI:** 10.1242/jeb.243867

**Published:** 2022-08-02

**Authors:** Camille Le Roy, Nicolas Silva, Ramiro Godoy-Diana, Vincent Debat, Violaine Llaurens, Florian Titus Muijres

**Affiliations:** 1 Department of Experimental Zoology, Wageningen University, 6709 PG Wageningen, The Netherlands; 2 Institut de Systématique, Evolution, Biodiversité (ISYEB), Muséum National d'Histoire Naturelle, CNRS, Sorbonne Université, EPHE, Université des Antilles, CP50, 75005 Paris, France; 3 Université Paris Cité, 12 rue de l’École de Médecine, 75006 Paris, France; 4 Laboratoire de Physique et Mécanique des Milieux Hétérogènes (PMMH, UMR 7636), CNRS, ESPCI Paris Université PSL, Sorbonne Université, Université de Paris Cité, 75005 Paris, France

**Keywords:** Animal locomotion, Evasive manoeuvres, Ecological specialisation, Insect flight, Wingbeat kinematics

## Abstract

Habitat specialization can influence the evolution of animal movement in promoting divergent locomotor abilities adapted to contrasting environmental conditions, differences in vegetation clutter or predatory communities. While the effect of habitat on the evolution of locomotion and particularly escape performance has been well investigated in terrestrial animals, it remains understudied in flying animals. Here, we investigated whether specialization of *Morpho* butterfly species into different vertical strata of the Amazonian forest affects the performance of upward escape flight manoeuvres. Using stereoscopic high-speed videography, we compared the climbing flight kinematics of seven *Morpho* species living either in the forest canopy or in the understory. We show that butterflies from canopy species display strikingly higher climbing speed and steeper ascent angle compared with understory species. Although climbing speed increased with wing speed and angle of attack, the higher climb angle observed in canopy species was best explained by their higher body pitch angle, resulting in more upward-directed aerodynamic thrust forces. Climb angle also scales positively with weight-normalized wing area, and this weight-normalized wing area was higher in canopy species. This shows that a combined divergence in flight behaviour and morphology contributes to the evolution of increased climbing flight abilities in canopy species.

## Abstract (precis)

**Summary:** Quantification of climbing flight kinematics among closely related butterfly species living in different strata reveals contrasted climbing flight ability, probably resulting from divergent flight behaviour and morphology.

## INTRODUCTION

The evolution of animal locomotion is mainly driven by selection on the ability to perform fitness-related tasks such as escaping from predators, finding mates or defending a territory (Alexander, 2013; Swingland and Greenwood, 1983). Habitat characteristics shape the selective regime acting on the evolution of animal movements, by generating physical constraints on animal motions (Crall et al., 2015; Goodman et al., 2008; Sathe and Husak, 2015) and by determining the community of species interacting with the studied animals (Denno et al., 2005; Willmott et al., 2017; Willson, 1974). Predation, in particular, is a strong selective pressure acting on the evolution of motion capacity, promoting, for example, high sprint speed or bursts of acceleration in prey species (Domenici et al., 2011; Irschick and Losos, 1999). The interactive effect of predation and physical characteristics of the habitat may result in trade-offs between traits enhancing successful escape (e.g. maximum speed, acceleration) and those enabling the animal to handle physical cluttering and substrates (e.g. stability, manoeuvrability). In lizards, for example, sprint speed dramatically varies with substrate complexity and environment clutter (Losos and Sinervo, 1989; Sathe and Husak, 2015), so that contrasted escape behaviours might be promoted depending on habitat configuration (Irschick and Losos, 1999). Ultimately, specialization of prey species in a particular habitat can thus influence the evolution of escape ability through changes in the morphological traits and behaviours involved (Goodman et al., 2008).

While the influence of habitat specialization on escape behaviour has been widely investigated in terrestrial animals, particularly in lizards (Calsbeek and Irschick, 2007; Foster et al., 2015; Irschick and Losos, 1999; Kotler et al., 2001; Martin and López, 1995), only a handful of studies have addressed how habitat influences the evolution of escape ability in flying animals (but see Devereux et al., 2008; Lima, 1993). Most flying animals exhibit a large range of flight manoeuvres (Aldridge, 1987; Betts and Wootton, 1988; Dakin et al., 2018), probably enabling successful escape from different predators and in various situations. Some escape behaviours may nonetheless be particularly effective in a given habitat. For example, in the common starling, *Sturnus vulgaris*, individuals foraging in environments with high vegetation escape predators by flying close to the ground, whereas those foraging in open grass swards show steep and upward escape trajectories (Devereux et al., 2008). This suggests contrasted escape strategies depending on either predator communities (i.e. staying close to the ground may reduce detection by some predators) or the constraints imposed by habitat structure on manoeuvrability (i.e. steep take-off may be impeded by dense vegetation). The behavioural response to predation is a highly plastic trait, and the evolution of this plasticity is probably favoured by the variability of attacks and the heterogeneity of the environment. A long-term selection in different environmental conditions may nevertheless promote divergent evolution of specific escape behaviours across habitats. So far, no study has tested whether habitat selection may result in adapted escape behaviour and ability among flying species showing divergent habitat specialization (see Goodman et al., 2008, for an example in lizards).

In tropical forests, significant variation in vegetation density, light, wind and feeding resources occurs along vertical strata, representing distinct microhabitats housing different communities of predator and prey species (Pearson, 1971; Vieira and Monteiro-Filho, 2003; Willmott et al., 2017). Butterfly communities, in particular, differ strikingly among forest strata, with some species flying only near the forest floor, and others in the mid-understory or at the canopy level (Cespedes et al., 2015; DeVries et al., 1997, 2010). Vertically segregated butterflies may thus have to escape different ranges of predators encountered in contrasted environmental conditions (Willmott et al., 2017). Such variation in the microhabitat and associated predator community may therefore strongly influence the evolution of escape flight behaviour and performance in tropical butterflies.

In this study, we investigated variation in climbing flight performance among Neotropical butterfly species from the genus *Morpho*, found in sympatry. The different species studied here show a strong vertical segregation: in most *Morpho* species, individuals fly within the first forest strata in the understory, but there is a clade of species where individuals fly much higher, up to the canopy (DeVries et al., 2010; Michael, 1911). Differences in spatial configuration between forest strata (cluttered versus open environment) are thought to play an important role in the evolution of flight behaviour and wing shape in *Morpho* butterflies (Chazot et al., 2016; DeVries et al., 2010; Le Roy et al., 2021). Habitat-associated predation may also generate a major selective force, acting in particular on the evolution of escape flight behaviour. Understory species may indeed face a different predatory community to canopy species, which may exert a different type of selection on escape ability. Contrasted species of insectivorous birds – which are the main predators of butterflies (Pinheiro and Cintra, 2017) – are known to be segregated along forest strata (Pearson, 1971; Walther, 2002). Butterflies living in the understory may also be more prone to escape attacks from reptiles or mantids, hence promoting the evolution of specific flight abilities. The behaviours increasing escape efficiency may also depend on spatial configuration: for instance, the range of possible escape manoeuvres may be more limited in cluttered environments as compared with open ones. Finally, the gradual increase in wind velocity and variability from the forest ground to the canopy (Kruijt et al., 2000) may promote specific flight adaptation to wind in canopy species.

Divergent evolution of flight ability may occur through changes in flight behaviour (e.g. wingbeat and body kinematics), resulting in contrasted performance metrics (e.g. speed, turning angle). However, morphology also crucially determines the achievable flight performance range (Aldridge, 1987; Berwaerts et al., 2002; Dakin et al., 2018). Morphological changes may thus have favoured the divergent evolution of flight behaviour in *Morpho* species specialized in different forest strata. To assess the relative contribution of behaviour and morphology in the evolution of escape flight performance, we jointly investigated the variation in the kinematics of escape flight and that in the wing and body morphology of different *Morpho* butterfly species living either in the understory or in the canopy.

We specifically focused on upward-directed flight, a power-demanding flight manoeuvre crucial for evading predators (Alexander and Vogel, 2004; Møller, 2010). Using stereoscopic high-speed videography, we quantified the wing and body kinematics of evasive climbing flights among two canopy species and five understory species. We estimated climbing performance using flight speed and ascent angle, and examined how variation in wingbeat kinematics and morphology among species contributes to variation in these climbing flight performance metrics. By accounting for the closer relatedness of species specialized in the same microhabitat, the phylogenetic tests then allowed us to assess whether the observed differences in climbing performance may simply be consistent with neutral divergence among species or might also be influenced by contrasted selective pressures encountered in the different microhabitats.

## MATERIALS AND METHODS

### Sampling

**Fig. 1.**
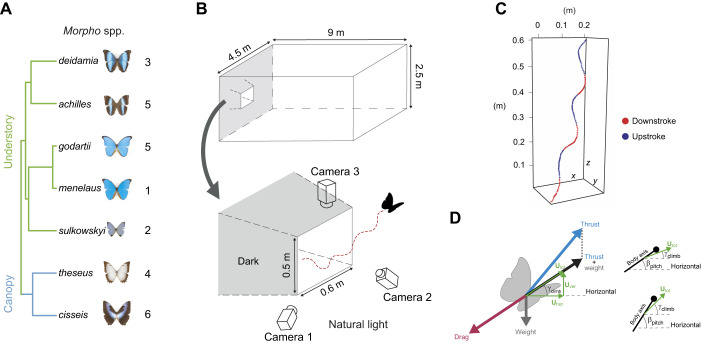
**Studied species and experimental set up used to record evasive climbing flights.** (A) The studied *Morpho* butterfly species (and number of individuals) shown with their phylogenetic relationships. (B) Butterflies were released in the tunnel, and flew towards the bright exit. Using the three orthogonally positioned video cameras, we reconstructed the flight trajectories of escaping butterflies. (C) Butterfly body position during a typical flight trajectory. The start and end of each wing stroke were identified on the trajectories to allow estimation of climbing efficiency per wingbeat (a wingbeat cycle includes a downstroke and an upstroke). (D) Free-body diagram describing the forces generated by a climbing butterfly flying at constant speed (left) and relationships between body pitch and climb angle (right). **U**_hor_, **U**_ver_ and **U**_total_, horizontal, vertical and total flight velocity, respectively; γ_climb_, climbing angle; β_pitch_, body pitch angle.

Sampling was performed from July to September 2017 in North Peru near the city of Tarapoto, either along the Rio Shilcayo river (06°27′07″S, 76°20′47″W; ca. 450 m) or near the village of San Antonio de Cumbasa (06°24′24″S, 76°24′25″W; ca. 470 m). We studied 26 *Morpho* butterflies in total, including 10 individuals of two canopy species and 16 individuals of five understory species (Fig. 1A). All butterflies were captured with hand-held nets, and were transported to the insectary in entomological envelopes inside plastic boxes, next to a bottle of frozen water to prevent them from overheating. The less-encountered and harder to catch canopy species were captured using an elongated net-shaft in an open environment such as the middle of rivers or bottom of waterfalls, where they tend to fly down from the canopy to cross the open environment.

### Filming the escaping climbing flight manoeuvres

The climbing flight experiments were performed in a large outdoor insectary within a few hours of capture. The experimental setup consisted of an outdoor insectary (9×4×2.5 m) containing a 1 m long funnel-shaped tunnel, built from dark mesh (Fig. 1B). Each butterfly was individually released into the dark tunnel, and usually flew directly towards the bright exit. After exiting the tunnel, the butterflies rapidly turned upwards, and performed a climbing escape flight manoeuvre.

We positioned three high-speed cameras (GoPro Hero4 Black set at 240 frames s^−1^, spatial resolution of 848×480 pixels) close to the tunnel exit, allowing the upward flight manoeuvre to be filmed simultaneously with all three cameras (Fig. 1B). The close proximity of the cameras to the flying butterfly allowed a clear view of its wings and body movements.

From all recordings, we selected the ascending flight sequences in which the wingbeats were clearly visible from all camera views. This resulted in a total of 72 flight sequences, including 41 flights from the two canopy species (cumulating 106 wingbeats) and 31 flights from the five understory species (cumulating in 77 wingbeats). Per individual, we recorded on average 2.7±1.9 flight sequences (mean±s.d.), including 2.5±1.3 wingbeats per flight sequence; see Fig. S1 for sampling details.

After being filmed, each individual was killed and weighed with a resolution of 0.01 g, and its detached wings were photographed in standardized conditions. From the photographs, we measured the wing area using WingImageProcessor in MATLAB (available at http://biomech.web.unc.edu/wing-image-analysis/) for subsequent analysis of the effects of morphology on climbing flight performance.

### Quantifying climbing flight performance from body kinematics

The stereoscopic videos were calibrated in three steps. We first corrected for the image distortion due to the wide-angle settings of the GoPro cameras using Argus DWarp Python routine (Jackson et al., 2016). Sequences of the same flight were then manually synchronized using a reference video frame. The camera system was then calibrated with the direct linear transformation (DLT) technique (Hartley and Sturm, 1995) by digitizing an object of known length (here, a wand) moved throughout the space of interest. The wand tracking and DLT coefficient computation were performed in MATLAB R2019b (MathWorks Inc.) using the *DLTdv6* routine (Hedrick, 2008) and the *easyWand* routine (Theriault et al., 2014), respectively.

After calibration, we also used *DLTdv6* (Hedrick, 2008) to digitize the 3D positions of the butterfly at each video frame by manually tracking the body centre in each camera view. The positional data of the butterfly throughout the climbing trajectory were post-processed using a linear Kalman filter (Muijres et al., 2014), providing smoothed temporal dynamics of the position, velocity and acceleration of the body centroid. In addition, the start and end of each wingbeat were identified by manually digitizing the video frames at which the wing was at the highest upstroke position and the lowest downstroke position, thus transcribing the spatial and temporal position of each wing stroke along the flight trajectory (Fig. 1C).

Based on the positional and wingbeat data, we quantified the climbing flight kinematics for each wingbeat by determining eight parameters: the wingbeat frequency (*f*_wingbeat_=1/Δ*t*_wingbeat_, where Δ*t*_wingbeat_ is the duration of the wingbeat), the horizontal, vertical and total distance travelled during the wingbeat (Δ*X*_hor_, Δ*X*_ver_, Δ*X*_total_, respectively), the corresponding wingbeat-average flight speeds (*U*_hor_, *U*_ver_, *U*_total_, respectively), and the mean climb angle during the wingbeat (γ_climb_=atan(*U*_ver_/*U*_hor_) (Fig. 1D).

Subsequently, we quantified the climbing flight kinematics per individual as the mean values of all wingbeats measured for that individual. The wingbeat-based data were used to analyse how climbing flight kinematics varies between canopy species and understory species; the individual-based data were used to perform subsequent analyses of the effect of individual morphology on climbing flight performance.

### Quantifying the body orientation and wingbeat kinematics of climbing flights

We quantified the detailed body and wingbeat kinematics executed for a subset of the recorded climbing flights. Because acquiring these data is labour intensive, we limited this analysis to the climbing flights of two canopy species (*Morpho cisseis* and *Morpho theseus*) and two understory species (*Morpho achilles* and *Morpho sulkowskyi*), and digitized three wingbeats for each species, each taken from a different individual (Fig. S1, see also Fig. S3A,B).

We determined the wing movements using the manual stereoscopic video tracker *Kine* in MATLAB (Fontaine et al., 2009; Fry et al., 2003). The tracker was originally developed for studying flight dynamics of two-winged fruit flies, and thus we adapted it to allow for tracking both the forewings and hindwings of butterflies. To do so, we built wing models of both the forewing and hindwing of each species by digitizing the wing outline on high-resolution photographs of a specimen. Each wing model consisted of 41 2D coordinates representing the wing shape, with the known hinge and tip positions. For simplicity, these wing models were subsequently considered as rigid flat plates, although real butterfly wings endure substantial deformation during the wing stroke (Wootton, 1981).

**Fig. 2.**
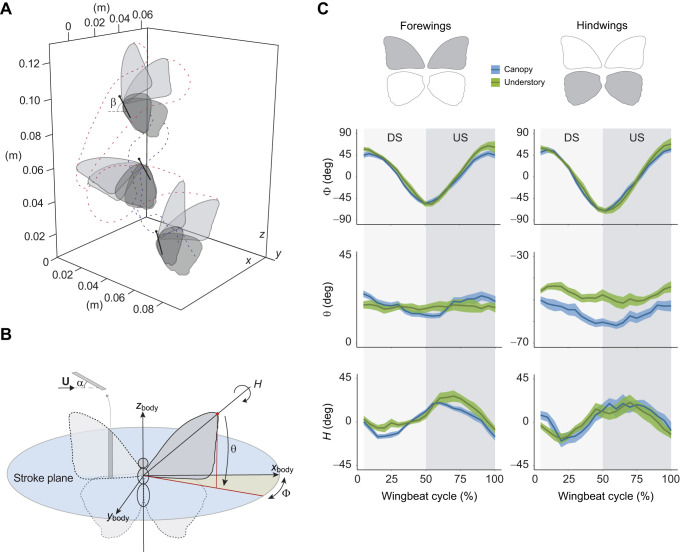
**Quantifying wingbeat kinematics of climbing flight.** (A) Example of a digitized wingbeat in an individual *Morpho cisseis*. Duration of the shown wingbeat is 0.26 s. Positions of forewing tips and hindwing tips throughout the wingbeat are indicated by the red and blue dotted lines, respectively (only three butterfly positions are shown for clarity). Body pitch (β) is shown for one butterfly position. (B) Conventional wing angles were measured at each time step *t*=0.004 s in the body reference frame. α, angle of attack; *H*, rotation angle; θ, deviation angle; ɸ, stroke angle; **U**, velocity. (C) Variation in the three wing kinematics angles throughout a wingbeat cycle. Traces indicate mean and 95% confidence interval at each time step for canopy and understory species. Values were averaged between the two forewings and the two hindwings. DS, downstroke; US, upstroke.

We used the manual tracker to quantify the position and orientation of the body and four wings in each consecutive video frame (Fig. 2A). Hereby, we defined the position and orientation of the body in the world reference frame, as a 3D position vector and the Euler angles, body yaw, pitch and roll. The angular orientation of the wings was expressed in the butterfly body coordinate system (Ellington, 1984a) (Fig. 2B), as Euler angles relative to the stroke plane: the stroke angle (Φ), deviation angle (θ) and rotation angle (*H*) of each wing (Fig. 2B,C). The stroke plane of each wing was defined as the plane normal to the long axis of the body passing through the hinge position of that wing. These hinge positions were kept constant throughout the wingbeat. The angle of attack (α) was computed as the angle between the wing plane and the velocity vector of the wing (Fig. 2B).

The three wing angles (stroke, deviation and rotation angle) were measured for each of the four wings independently (left and right forewing and hindwing) and then averaged between the two forewings and the two hindwings, separately (Fig. 2C). We estimated the translational speed and angle of attack throughout the wingbeat using a blade-element approach by dividing each wing into 10 elements along the wingspan. To quantify the wingbeat-specific angle of attack of each wing (α_wingbeat_), we extracted the blade-element average value at mid-stroke, where aerodynamic force production is close to maximum (Tian et al., 2013). We quantified the wingbeat-specific speed of the wing (*U*_wingbeat_) as the mean translational wing speed for all blade elements of all four wings throughout the wingbeat. We averaged the wing speed across the four wings because this speed was almost identical between the wings. Finally, based on the body kinematics, we determined the mean body pitch angle as the average angle between the body axis and the horizontal during the wingbeat (β_wingbeat_) (Fig. 2A). These reduced kinematics parameters allow a simplified description of the complex 3D wing movements that includes effects of body and wing deformations, but these summarized parameters are still relevant to characterize climbing flight kinematics of *Morpho* butterflies.

### Quantifying and studying climbing flight performance

Here, we define climbing flight performance using the two flight kinematics parameters flight speed *U*_total_ and climb angle (γ_climb_). We used these two parameters because combined they define the climb speed (Fig. 1D), and they can independently be controlled by a flying animal.

We studied the climbing flight in *Morpho* butterflies using two approaches. (1) The first (study 1) focused on testing how climbing flight dynamics and performance vary between canopy and understory species. This analysis was based on the body trajectory data of the complete dataset of all recorded flight sequences. (2) The second approach (study 2) focused on how variation in flight and wingbeat kinematics impacts climbing flight performance. This analysis relied on both the whole dataset of flight kinematics and the supplementary dataset where the full body and wingbeat kinematics was tracked.

### Testing how microhabitat affects climbing flight kinematics and performance

**Fig. 3.**
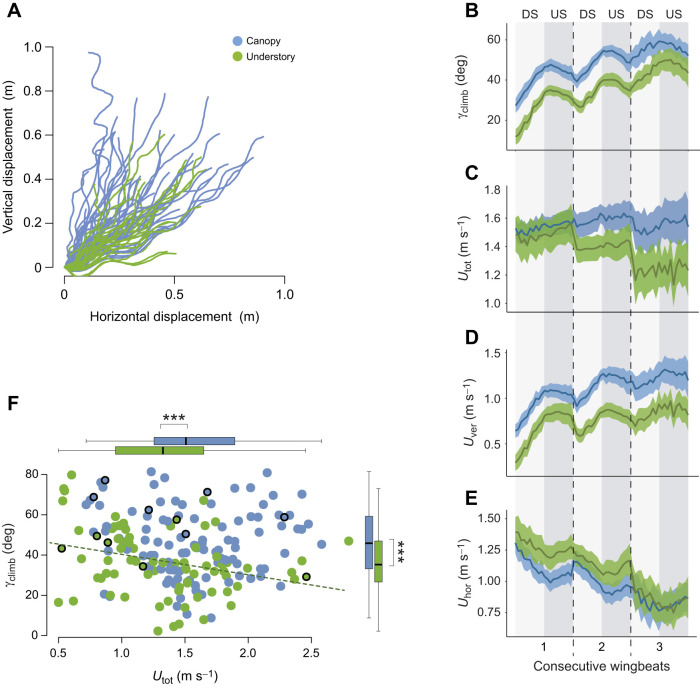
**Variation in climbing kinematics parameters between microhabitats.** (A) The 72 analysed flight paths viewed from the side (*N*=41 flights for canopy species; *N*=31 flights for understory species). (B–E) temporal dynamics of (B) climb angle (γ_climb_), (C) total flight speed (*U*_tot_), (D) vertical flight speed (*U*_ver_) and (E) horizontal flight speed (*U*_hor_) over three consecutive wingbeats. DS, downstroke; US, upstroke. Traces indicate mean and 95% confidence interval at each video frame (240 frames s^−1^). Time was normalized between wingbeats and between stroke phases. (F) Relationship between climb angle and flight speed (each point is one wingbeat). A negative correlation between these two metrics was found in understory butterflies only (*r*=0.31, *P*=0.006; regression line shown). Results of ANOVA testing the effect of microhabitat on climb angle and flight speed are indicated by asterisks (***P<0.001) beside the boxplots (median, upper and lower quartiles and 1.5× interquartile range). The wingbeats for which detailed wing and body movements were quantified (see Fig. 2) are highlighted with black circles.

Analysis of the climbing flight dynamics and performance consisted of two parts (study 1a and 1b). First, we studied how climbing flight performance differed between butterflies from the two microhabitats by analysing the dynamics of the climbing performance metrics flight speed and climb angle (study 1a) (Fig. 3). Second, we studied climbing flight kinematics in more detail by analysing how the eight flight kinematics parameters differed between the canopy and understory butterflies using ANOVA (study 1b).

To analyse the dynamics of the climbing performance (study 1a), we first determined the temporal dynamics of flight speed and climb angle throughout the climbing flight manoeuvres of understory and canopy butterflies, separately. For this, we aligned the consecutive wingbeats of all recorded climbing flights, and then determined the average temporal dynamics of flight speed and climb angle for each microhabitat (Fig. 3B–E).

To further compare climbing flight performance among microhabitats (study 1b), we tested how the eight flight kinematics parameters differed between understory and canopy butterflies, using a set of ANOVA, and a MANOVA on all parameters combined. Regular ANOVA were used to test the effect of microhabitat while controlling for species, individual, flight sequence and wingbeat number within the flight sequence (first, second, third wingbeat). The dataset used was composed of *N*=106 wingbeats for the canopy species, and *N*=77 wingbeats for the understory species. Phylogenetic ANOVA were used to test for differences between microhabitats while controlling for the phylogenetic distance between species (Freckleton et al., 2002), based on the phylogeny of Morpho butterflies established in Chazot et al. (2016) (Fig. 1A). The dataset for the phylogenetic ANOVA consisted of averaged values per species, with *N*=2 canopy species and *N*=5 understory species.

### Studying how variations in body and wingbeat kinematics impact climbing flight performance

Analysis of how flight and wingbeat kinematics impact climbing flight performance also consisted of two parts (study 2a and 2b). First, we studied how the wingbeat kinematics varied between the understory and canopy species (study 2a). Second, we studied how variation in either flight or wingbeat kinematics on the one hand and morphology on the other hand affected climbing flight performance, based on an aerodynamic model of a flying butterfly (study 2b).

Hereby, we characterized climbing flight performance using climbing flight speed (*U*_total_) and ascent angle (γ_climb_). A flying animal can increase its climbing flight speed by enhancing the aerodynamic thrust magnitude, and it can maximize the climb angle by directing the aerodynamic thrust vector more upwards (Fig. 1D).

To study how *Morpho* butterflies control their climbing flight speed (*U*_total_), we applied an aerodynamic thrust force model to a climbing butterfly, as described in Ellington (1984b), using the equation: 
(1)

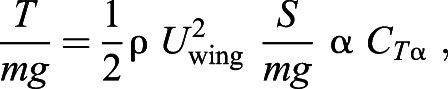
where *T*/*mg* is the weight-normalized aerodynamic thrust force produced during a wingbeat. This normalized force varies quadratically with the speed of the beating wing (*U*_wing_), and linearly with air density (ρ), the weight-normalized wing area (*S*/*mg*), the wing angle of attack (α) and the angle of attack-specific thrust coefficient (*C_T_*_α_). Note that the weight-normalized wing area equals the inverse of wing loading. Here, we normalized the aerodynamic thrust with weight (*mg*) because flight accelerations scale with this ratio, and thus climbing speed should increase with this weight-normalized aerodynamic thrust force. This weight equals the product of body mass *m* and the gravitational acceleration scalar *g*.

Thus, to test how much kinematics control climbing flight speed, we used a set of linear regressions to correlate the climbing speed (*U*_total_) with the three kinematics and morphology metrics in Eqn 1 (*U*_wing_, *S*/*mg* and α_wingbeat_).

To study how *Morpho* butterflies control their ascent angle (γ_climb_) during climbing flight, we took the orientation of the aerodynamic thrust force vector into consideration (Fig. 1D). Flying animals can modify the direction of this force vector through variation in the angle of attack of the beating wing (α_wingbeat_) and in the body pitch angle (β_wingbeat_) (Fig. 1D).

Behavioural adjustments in angle of attack will result in different lift and drag forces produced by the wings, causing an adjustment of the aerodynamic force vector relative to the body orientation. In contrast, a change in the body pitch angle reorients the thrust vector relative to the world reference frame, without changing the force vector relative to body orientation. This last mechanism is commonly used by insects as a way to control their forward flight speed (David, 1978) and to perform banked turns (Muijres et al., 2014). Because this body-fixed force vectoring is similar to how helicopters are steered, this mechanism is often referred to as the helicopter model for insect flight control (David, 1978; Dickinson and Muijres, 2016).

A flying butterfly needs to adjust its body pitch angle via changes in its wingbeat pattern, and so the body pitch angle itself is not directly a flight control variable. Therefore, here we used body pitch angle as a flight kinematics parameter that quantifies the functional mechanism used by butterflies for reorienting of the thrust vector, and not a control variable itself.

Based on this thrust force vector model, we tested how *Morpho* butterflies controlled their climb angle using a set of linear regressions, where we correlated the ascent angle (γ_climb_) with the two relevant kinematics variables, the angle of attack of the beating wing (α_wingbeat_) and the body pitch angle (β_wingbeat_). Using the same approach, we also tested how the corresponding morphology metric weight-normalized wing area (*S*/*mg*) affected climb angle.

## RESULTS

### Canopy species exhibit a higher climbing flight performance

In study 1, we tracked the climbing flight trajectories in the whole dataset: we studied 106 wingbeats in 41 flights of 2 canopy species and 77 wingbeats in 31 flights of 5 understory species (Fig. 3A; Fig. S1). There was a high variability in most of the measured parameters (Table 1). We found a significant inter-individual variation, and for a given individual, contrasted performance among the different flights it performed could also be observed. Climbing performance also varied between consecutive wingbeats within a flight sequence performed by an individual, attesting to the changes in the flight dynamics over the climbing trajectory. Despite such variation, we nevertheless detected an effect of the butterfly microhabitat on several aspects of the dynamics of climbing performance.

**Table 1.**
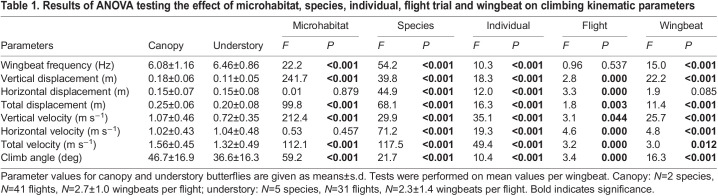
Results of ANOVA testing the effect of microhabitat, species, individual, flight trial and wingbeat on climbing kinematic parameters

Focusing on the climb performance metrics (study 1a) climb angle and climbing speed, we found that butterflies from canopy species tended to climb both steeper and at higher speed as compared with understory butterflies (Fig. 3A–D). Our statistical analysis of these flight performance metrics shows that canopy butterflies climb at 28% higher ascent angles than understory ones (Fig. 3F, canopy species: γ_climb_=46.7±17 deg; understory species: γ_climb_=36.5±16 deg, *P*<0.001). During these climbing phases, canopy butterflies flew 18.2% faster than understory butterflies (Fig. 3F, canopy species: *U*_total_=1.56±0.56 m s^−1^; understory species: *U*_total_=1.32±0.49 m s^−1^, *P*<0.001). Noticeably, for the same climb angle of 50 deg, canopy butterflies flew on average 25% faster than understory butterflies (canopy species at γ_climb_=50 deg: *U*_total_=1.55±0.51 m s^−1^; understory species at γ_climb_=50 deg: *U*_total_=1.24±0.40 m s^−1^). The distribution of climb angles and climbing flight speeds (Fig. 3F) shows that canopy species tended to climb simultaneously at high ascent angles and at high flight speeds; in contrast, understory species tended to climb at either a high ascent angle or a high flight speed, suggesting that there is a trade-off between these two performance metrics in understory species (correlation between climb angle and flight speed in canopy butterflies: *r*=−0.04, *P=*0.64; in understory butterflies: *r=*−0.31, *P*=0.006) (Fig. 3F).

The temporal dynamics of the climb angle shows that climb angle increased throughout the consecutive wingbeats within each climbing manoeuvre (Fig. 3B). While this was strikingly similar in the canopy and understory butterflies, there was a continuous offset between the two types of species, whereby the climb angle observed in canopy butterflies was systematically higher.

In contrast, the temporal dynamics of flight speed was quite different between canopy and understory butterflies (Fig. 3C). For both groups, flight speed slightly increased over the course of each wingbeat and dropped in-between wingbeats. But the speed losses between wingbeats were markedly larger in understory than in canopy species. As a result, butterflies from the canopy maintained – if not increased – their flight speed while climbing, whereas butterflies from the understory decelerated while climbing.

A flying animal can increase its climbing angle by increasing the vertical flight speed, or decreasing the horizontal flight speed. The vertical climb speed observed in the canopy species was 49% higher than in the understory species (canopy species: *U*_ver_=1.07±0.46 m s^−1^; understory species: *U*_ver_=0.72±0.35 m s^−1^, *P*<0.001), whereas the horizontal speed did not differ significantly between understory and canopy species (*U*_hor_: *P*=0.42; Fig. 3D,E). This shows that canopy butterflies reach higher climb angles primarily because of greater vertical climb speed. This is further supported by the striking similarities in the temporal dynamics of the vertical flight speed and of the corresponding climb angle (Fig. 3D and B, respectively).

The multivariate statistical analysis applied to all climbing flight parameters (study 1b) shows a significant effect of microhabitat on climbing parameters (MANOVA: Wilks' λ=0.72; *P<*0.001; *N*=183 wingbeats), confirming the greater climbing performance observed in canopy species as compared with understory ones. See also Fig. S3A for a principal component analysis performed on all the climbing parameters. These differences were no longer significant when controlling for phylogenetic distance (Table S1). The effect of selection across microhabitats could thus not be distinguished from the phylogenetic divergence, probably as a result of the low number of species studied, limiting the statistical power.

### Wingbeat kinematics during climbing flight of *Morpho* butterflies

We then tracked the body and wingbeat kinematics in a subset of these butterflies from two canopy and two understory species (study 2). For each of these four species, we digitized three wingbeats, each taken from a different individual. Based on these data, we reconstructed the average wingbeat kinematics for the understory butterflies and canopy butterflies separately (Fig. 2).

This detailed study 2a on wingbeat kinematics revealed several differences between canopy and understory species (Fig. 2C; Fig. S2). Most apparently, the deviation angle of the hindwings differed between groups: canopy butterflies deviated their hindwings further from the stroke plane than understory butterflies during both the upstroke and the downstroke (hindwings in canopy species: θ=−56±4 deg; hindwings in understory species: θ=−48±3 deg, *P*=0.01; Fig. S2). Also, the forewings of understory butterflies seem to operate at slightly higher wing stroke amplitudes and wing rotation angles, but these differences were not significant (wingbeat amplitude in canopy species: *A*_φ_=114±19 deg; in understory species: *A*_φ_=128±26 deg, *P*=0.34).

### Climbing performance depends on both wingbeat kinematics and morphology

**Fig. 4.**
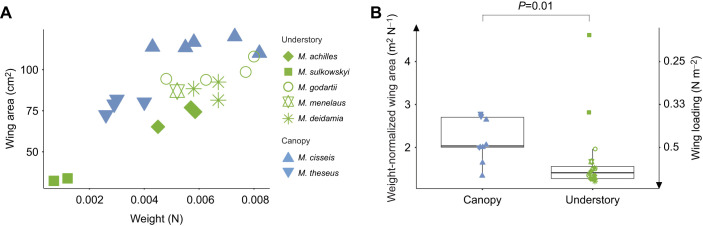
**Difference in wing area and body weight among the studied butterflies.** (A) Relationship between wing area and weight in canopy and understory species. (B) Difference in weight-normalized wing area between canopy and understory butterflies. Note that weight-normalized wing area is the inverse of wing loading, as shown on the right axis. Statistical differences were tested using Wilcoxon rank sum tests.

We consequently studied how variation in wingbeat kinematics and morphology affects climbing performance in *Morpho* butterflies (study 2b), using our aerodynamic model for climbing flight performance (Eqn 1). For this, we quantified the body and wing morphology of all individuals used in the flight datasets (*N*=10 individuals from two canopy species and *N*=16 individuals from five understory species) (Fig. 4), and the detailed body and wingbeat kinematics of two canopy species (*Morpho cisseis* and *Morpho theseus*) and two understory species (*Morpho achilles* and *Morpho sulkowskyi*). The resulting wingbeat kinematics dataset consisted of 12 digitized wingbeats, each from a different individual, and three individuals per species (Figs 2 and 3F).

We focused on the weight-normalized wing area *S*/*m**g***, as a metric of butterfly morphology, because of its high relevance in climbing flight performance (Eqn 1) (Fig. 4). This morphological parameter was 28% higher in canopy butterflies than in understory species (canopy species: *S*/*m**g***=2.24±0.51; understory species: *S*/*m**g***=1.74±0.88, *P*=0.01; Fig. 4B), suggesting that canopy species have a higher potential for producing weight-normalized thrust forces (Eqn 1). The relative distribution of wing area versus weight (Fig. 4A) shows that the higher *S*/*m**g*** observed in canopy species primarily stems from a greater wing area. Wing area was indeed significantly larger in the canopy species as compared with the understory species (canopy species: wing area 100.42±19.27 cm^2^; understory species: wing area 79.28±21.15 cm^2^, *P*=0.01), whereas butterfly weight did not differ between canopy and understory species (*P*=0.53).

Our body and wingbeat kinematics analysis was based on a subset of 12 wingbeats from 12 different individuals (highlighted in Fig. 3F). Because of this reduced sample size, we first tested how the relevant flight kinematics metrics differed between the total dataset and the subset of 12 wingbeats (Fig. S3). This showed similar trends in the two datasets (Fig. S3), whereby the canopy species flew faster, climbed steeper, travelled further per wingbeat and had a lower wingbeat frequency than the understory species. All these differences were significant for the complete dataset (*N*=183 wingbeats), but for the reduced subset (*N*=12 wingbeats), only climb angle was significantly higher in the canopy species than in the understory species.

Thus, because of its reduced sample size (*N*=12 wingbeats), we were unable to use the wingbeat kinematics dataset to quantitatively compare the wingbeat kinematics between canopy and understory species (study 2a). Instead, we used the wingbeat kinematics dataset primarily to study the functional effect of wingbeat kinematics on climbing flight performance in *Morpho* butterflies in general (study 2b). This analysis was based on the aerodynamic thrust force model for flapping flight (Eqn 1), and should allow us to determine how *Morpho* butterflies adjust their body and wingbeat kinematics to vary their climbing flight performance.

**Fig. 5.**
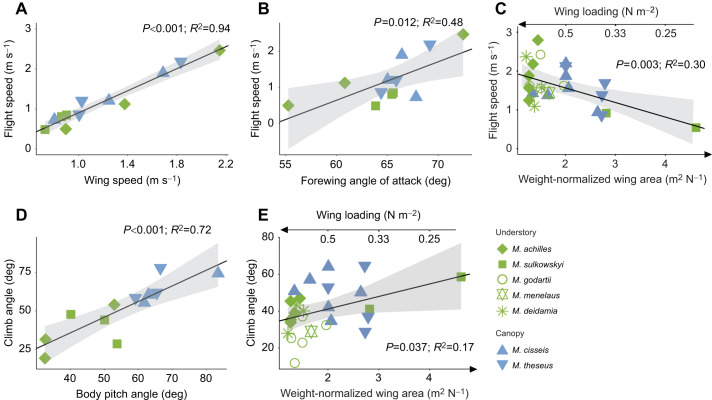
**Effect of wingbeat kinematics and weight-normalized wing area on flight speed and climb angle.** (A–C) Flight speed increases with wing speed (A) and forewing angle of attack (B), but decreases with weight-normalized wing area (C). (D,E) Climb angle increases with body pitch angle (D) and with weight-normalized wing area (E). Grey traces are the 95% confidence interval. The effect of wing kinematics was tested on a subsample (*N*=12 individuals), whereas the effect of morphology was tested on the full sample (*N*=26 individuals) (see Fig. S1 for sample details). The results of linear regression are shown on each plot (*P*-value and *R*^2^).

Using this approach, we first tested how flight speed varied with the relevant kinematics and morphology metrics identified in Eqn 1 (Fig. 5A–C, Table 2). This showed that flight speed increased linearly with flapping wing speed (Fig. 5A; *R*²=0.94, *F*_10,0.16_=157.6, *P*<0.001) and forewing (but not hindwing) angle of attack (Fig. 5B; *R*²=0.48, *F*_10,0.49_=9.18, *P*=0.012). Flight speed also decreased linearly with weight-normalized wing area (*S*/*m**g***) (Fig. 5C; *R*²=0.30, *F*_24,0.45_=10.47, *P*=0.003), suggesting that canopy butterflies (showing higher *S*/*m**g***) may have lower flight speed, contradicting the previously found higher climb speed in canopy species. A negative correlation between *S*/*m**g*** and flight speed (Fig. 5C) is however expected here because forward (horizontal) flight speed scales with wing loading (the inverse of *S*/*m**g***), and understory butterflies tend to have higher horizontal flight speed (Fig. 3E). As noted above, because of the low sample size of the studied wingbeat subset (study 2), these data do not allow differentiation between these effects in canopy and understory species.

**Table 2.**
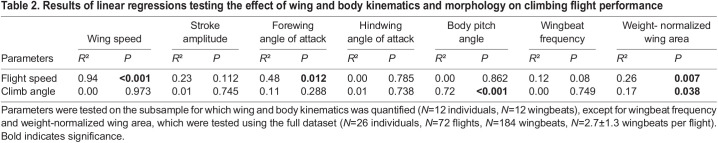
Results of linear regressions testing the effect of wing and body kinematics and morphology on climbing flight performance

Second, we tested how climb angle varied with the functionally relevant kinematics and morphology metrics angle of attack, body pitch angle and weight-normalized wing surface area (Fig. 5D,E, Table 2). This showed that climb angle significantly correlates with body pitch angle and weight-normalized wing area (Fig. 5D,E). In contrast, we detected no effect of wing angle of attack on the climb angle (forewings: *P=*0.28; hindwings: *P=*0.73).

Wingbeat-average climb angle was positively correlated with body pitch angle (Fig. 5D; *R*²=0.72, *F*_10,9.88_=25.55, *P*<0.001). In addition, the wingbeat-average body pitch angle was a markedly 50% higher in canopy species than in understory species (canopy species: β_wingbeat_=67±9 deg; understory species: β_wingbeat_=44±10 deg, *P=*0.001), (Fig. 5D). The increased climbing performance observed in canopy species is thus mostly due to differences in body orientation, rather than wingbeat kinematics. This suggests that steeper climbing individuals do not produce a larger thrust force, but instead that butterflies direct their aerodynamic thrust vector more upwards by increasing their body pitch angle.

These combined results thus show that the higher climb performance observed in canopy species is due to differences in both flight behaviour and morphology, as the higher climb angles exhibited in canopy butterflies are jointly enhanced by their higher body pitch angle and weight-normalized wing area.

## DISCUSSION

### Climbing flight performance depends on both body and flight kinematics

In this study, we investigated the influence of microhabitat specialization on the evolution of climbing flight performance in *Morpho* butterflies. We showed that in canopy species, climbing speed and ascent angle are on average higher than those of understory species. The dynamics of climbing flight manoeuvres was markedly different between canopy and understory butterflies: while overall velocity was similar in the two groups, canopy butterflies maintained and built on this velocity through subsequent wing strokes, resulting in a fast and steep climbing trajectory. In contrast, butterflies from understory species lost velocity as they climbed, and had trajectories with less steep slopes. This suggests a higher climbing performance in canopy species, which could allow these canopy butterflies to perform better upward escape flight manoeuvres.

To understand how canopy butterflies achieve this higher climbing performance, we characterized the detailed wing and body kinematics of the climbing flight manoeuvres of *Morpho* butterflies, and tested how those kinematics affected ascent angle and climbing flight speed. Using an aerodynamic model-based approach (Eqn 1), we showed that flight speed scaled positively with both the speed of the flapping wings and the mean wingbeat angle of attack of the forewings, but not of the hindwings. Wing speed and angle of attack were however comparable among butterflies from canopy and understory species. The higher climbing flight speeds measured in canopy species thus cannot be attributed to a difference in wingbeat kinematics. Wing movements should be measured on a larger sample of butterflies to ascertain the role of wingbeat kinematics in the contrasted climbing performance of canopy and understory species. Interestingly, the positive effect of wing angle of attack on climbing speed was found for the forewings only, supporting a prominent role of forewing movement during butterfly flight (Jantzen and Eisner, 2008; Le Roy et al., 2019). The angular orientation of the wings (stroke, deviation and rotation angle) was also broadly similar between the two groups, albeit individuals from canopy species deviated their hindwings further from the stroke plane than those from understory species. This subtle hindwing movement might play a role in the enhanced climbing flight ability of the canopy species.

In contrast to the wing kinematics, body orientation during climbing flight was strikingly different between canopy and understory species. Butterflies from canopy species showed markedly higher body pitch angles, which resulted in an increased ascent angle. Thus, while similar wing kinematics are used during climbing flight in butterflies inhabiting the canopy and the understory, canopy butterflies achieve a higher climbing performance by directing aerodynamic force further upward via higher body pitching. The increased climbing performance observed in canopy species thus stems primarily from changes in the orientation of the aerodynamic force vector produced by the flapping wings, and less so from the aerodynamic force magnitude. These results show that flights observed in *Morpho* butterflies are generally consistent with the predictions brought by the so-called helicopter model for insect flight, where the aerodynamic thrust force vectoring is primarily achieved via body rotations, rather that adjustments in the wingbeat kinematics. This helicopter model was also shown to be relevant for understanding flight control and manoeuvrability in other natural flyers, including flies and birds (David, 1978; Muijres et al., 2014; Ros et al., 2011). Overall, these results emphasize the importance of studying both wing and body kinematics to identify divergent flight abilities between species.

The wingbeat kinematics parameters used in this study were estimated using simplified rigid blade-element models of the forewings and hindwings. More sophisticated wing models are now needed to study the climbing flight of *Morpho* butterflies in more detail; for example, when studying flight control or the effect of wing deformation on climbing flight performance (Wootton, 1981).

The high variability in climbing performance measured here among and within individuals calls for further quantification of more individuals to strengthen our findings. Nonetheless, such variability attests to the large performance spectrum achievable with a given morphology. Our data thus show that variation in wing and body movement in an individual butterfly can result in strikingly different climbing performance. This highlights the remarkable plasticity of flight, but in turn makes it challenging to distinguish differences in behaviour (motivation to fly up) from those of performance capacity (Losos et al., 2002).

### The contribution of morphology to climbing flight performance

Selective pressures promoting specific escape abilities in different habitats may ultimately result in morphological changes, altering the limit imposed by morphology on performance (Garland and Losos, 1994; Wainwright, 1991). The contrasted climbing performance observed among *Morpho* species may thus result from and/or be enabled by divergent morphology.

We investigated this hypothesis by focusing on the ratio of wing area and body weight (*S*/*m**g***), which is the inverse of wing loading *W*/*S* and a parameter strongly constraining flight ability and particularly manoeuvring capacity (Norberg, 2002; Srygley and Dudley, 1993). Larger weight-normalized wing area (high *S*/*m**g***, characterizing the canopy species) limits forward flight speed but in turn enables the execution of sharper turns (Aldridge, 1987; Norberg, 2002). In contrast, lower weight-normalized wing area (low *S*/*m**g***, mostly characterizing the understory species) is generally associated with higher horizontal flight speeds required for weight support (Norberg, 2002).

Using our aerodynamic model for climbing butterfly flight (Eqn 1), we identified this weight-normalized wing area as also relevant for climbing flight performance. When testing this, we found a positive effect of *S*/*m**g*** on climb angle, suggesting that the steeper climb angle observed in canopy species may be promoted by their morphology. Indeed, high values of *S*/*m**g*** enable slower forward flight and increase body responsiveness, thereby permitting quick changes in body orientation. In understory species, lower *S*/*m**g*** limits the ability to fly slowly and hence might prevent them from achieving a steep climb angle. Accordingly, forward flight speed has been shown to be higher in understory *Morpho* species (Le Roy et al., 2021). The higher climbing efficiency observed in canopy species may thus not only stem from specific flight kinematics, as their divergent morphology also makes sharp upward turns easier to perform. This result suggests that microhabitat specialization does not just result in behavioural variation (i.e. in differences in the tendency or motivation to fly up) but rather implies the joint divergent evolution of morphological and behavioural traits.

### Selective forces favouring higher climbing efficiency in canopy species

Although the difference in climbing performance was striking among microhabitats, the small number of species studied here limits our capacity to disentangle the relative influence of phylogenetic distance and microhabitat selection on the evolution of this trait. Contrasted selective pressures exerted by microhabitats might nevertheless influence the evolution of climbing flight performance in *Morpho* species. Fast and steep climbing flight is probably advantageous when escaping ground predators and may therefore be under stronger selection in butterflies living in the understory, which probably face more frequent attacks coming from the ground. Surprisingly, our study described a less efficient climbing flight in understory species than in canopy species. However, the physical structure of the habitat might select for different escape behaviours. Butterflies living in the dense vegetation of the understory may face stronger physical constraints (e.g. branches and leaves) impeding the execution of upward escape flight, compared with butterflies mostly flying in the open environment of the canopy. Fast and steep ascending flight, which is among the most efficient aerial escape tactics (Hedenström and Rosén, 2001), may thus be promoted in canopy species because most of the time this flight manoeuvre is achievable in an open habitat. In agreement with this hypothesis, a survey of birds' escape tactics suggests that species living in open landscape are more prone to using climbing flight to out-compete aerial predators (Lima, 1993). Climbing escape manoeuvres are moreover particularly effective in prey for evasion of large aerial predators (Hedenström and Rosén, 2001), which are mainly insectivorous birds for *Morpho* butterflies (Pinheiro and Cintra, 2017).

Finally, the evolution of climbing flight ability is also probably influenced by ecological factors other than escape from predators. All *Morpho* species are known to feed on decaying fruits falling on the forest floor (Blandin, 1988; Young, 1972). Food acquisition may constrain individuals from canopy species to go down in the understory to search for rotting fruits. They are also occasionally observed mud-puddling on river banks: river mud is a key mineral resource for butterflies, and this behaviour is widespread in many species (Beck et al., 1999), especially in tropical forests, where mineral resources are limited. They may therefore use climbing flight to a large extent to fly back to the canopy. Flying up to the canopy might also benefit those individuals because it allows access to an open environment where extended gliding flight periods are possible and greatly reduce the energy costs associated with flight (Le Roy et al., 2021). In contrast, individuals from understory species may use climbing to a lesser extent as they typically fly closer to the forest floor, and may also be less able to cope with a windy environment (Combes and Dudley, 2009). Further ecological and behavioural studies on *Morpho* butterflies are critical to assess how variation in ecological traits, such as escape tactics or foraging behaviours, may influence the evolution of their flight behaviour and performance.

## Supplementary Material

Supplementary information
