# Supplementary material for: Divergence of climbing escape flight performance in Morpho butterflies living in different microhabitats
Source: J Exp Biol. 2022 Aug 2;225(15):jeb243867. doi: 10.1242/jeb.243867 (PMC9440751; doi:10.1242/jeb.243867)
Supplement: Supplementary information [file jexbio-225-243867-s1.pdf]

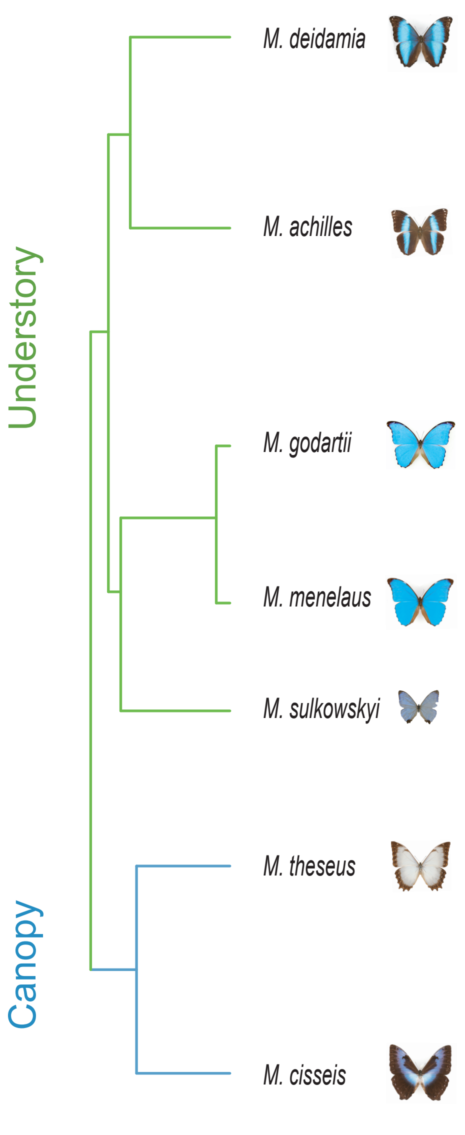

Species	Individuals		Nb of flights	Mean nb of wingbeats per flight
M. deidamia	3	Ind. #1	1	3
		Ind. #2	1	1
		Ind. #3	1	1
M. achilles	5	Ind. #1 ★	2	1.5
		Ind. #2	1	1
		Ind. #3	1	3
		Ind. #4 ★	1	2
		Ind. #5 ★	3	2.7
M. godartii	5	Ind. #1	2	1
		Ind. #2	1	1
		Ind. #3	2	2
		Ind. #4	3	2
		Ind. #5	3	2
M. menelaus	1	Ind. #1	3	2
M. sulkowskyi	2	Ind. #1 ★	5	5
		Ind. #2 ★	1	6
M. theseus	4	Ind. #1 ★	8	2.25
		Ind. #2 ★	4	3.5
		Ind. #3 ★	1	5
		Ind. #4	4	3.75
M. cisseis	6	Ind. #1	2	2
		Ind. #2 ★	7	2.3
		Ind. #3 ★	5	2.6
		Ind. #4 ★	3	2.3
		Ind. #5	4	2
		Ind. #6	3	2

★ Individuals for which wing and body movement was quantified during one of their wingbeats.

Fig. S1. Sampling details. The studied species are shown with their phylogenetic relationships on the left. Number of individuals per species, flights per individual, and mean number of wingbeats per flight are shown in columns. Red stars indicates the individuals for which wingbeat kinematics was quantified (see Fig. 2).

Journal of Experimental Biology • Supplementary information

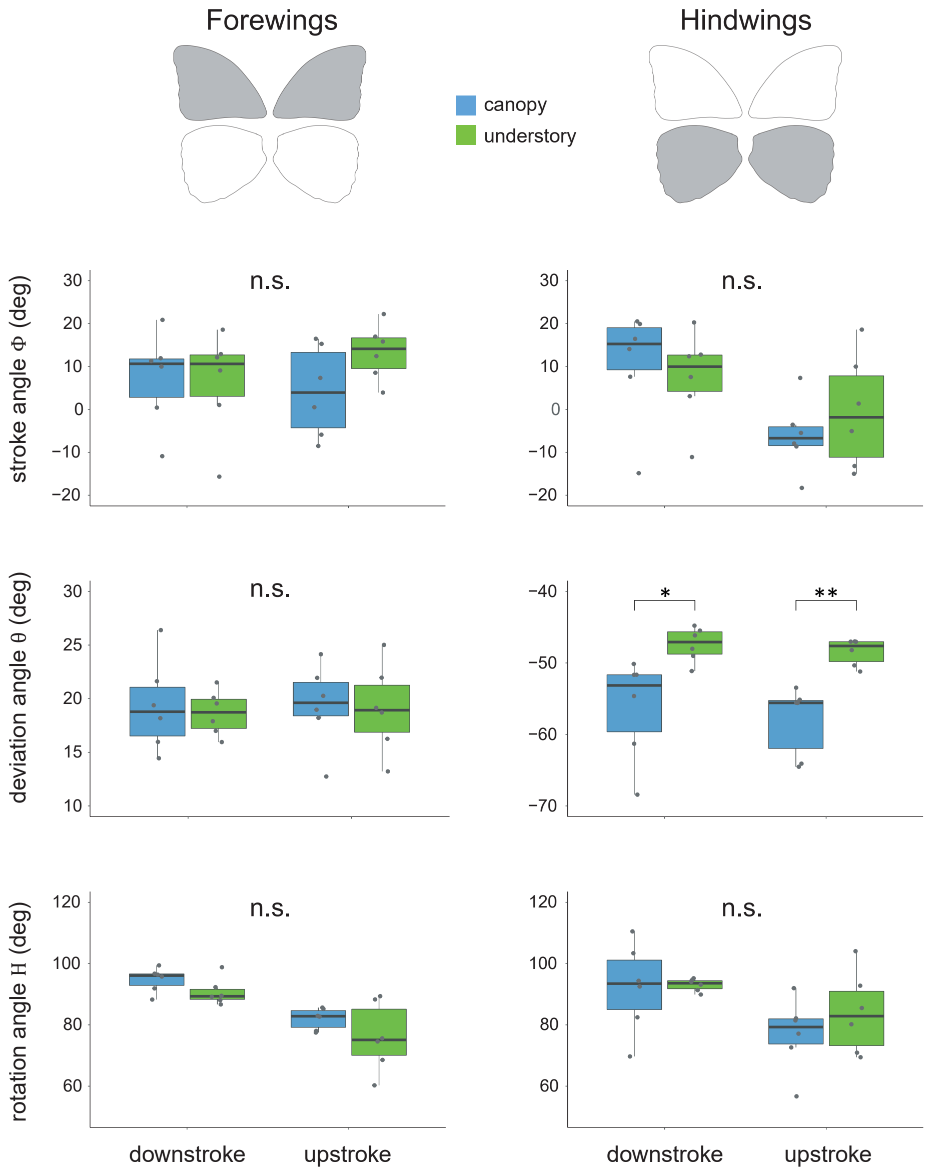

Fig. S2. Variation in mean values of wing angles among microhabitats. Mean values of wing angle were measured separately one the forewings (left column) and the hindwings (right column) using the averaged value of left and right wings for each wing pair. For each wing pair, the mean value of wing angle was moreover measured separately in the down- and upstroke phase (left and right respectively within each panel). Statistical difference between canopy and understory were tested using ANOVAs (*$p<0.05$; **$p<0.01$; n.s.= not significant).

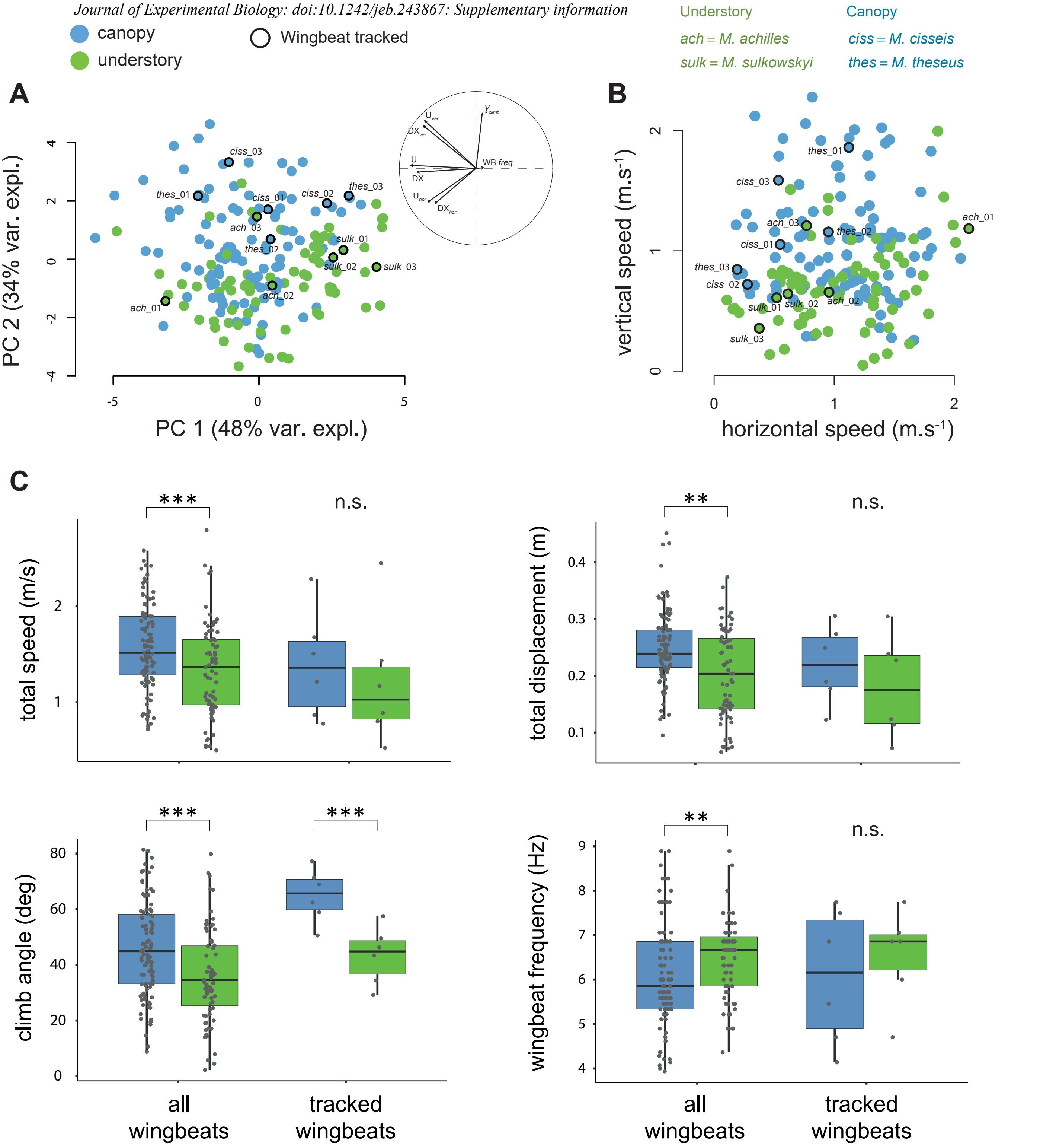

Fig. S3. Comparison of climbing performance between the full and the sub-dataset. (**A**-**B**) Wingbeats for which detailed wing and body movement was quantified (see Fig. 2) are highlighted with black circles (sub-dataset). Only the position of the body centre was tracked for the other wingbeats (full dataset) (see Fig. 1C). The tracked wingbeats are shown on Principal Component Analysis performed on the set of 8 flight parameters (**A**) and on a plot of vertical *vs*. horizontal speed (**B**). Panel **C** shows comparisons between the full and the sub-dataset for the different climbing performance metrics. One point is one wingbeat. The full dataset include 4 flights from 2 canopy species (totalizing 106 wingbeats), and 31 flights from 5 understory species (totalizing 77 wingbeats). The sub-dataset includes 2 canopy and 2 understory species, in which three wingbeats were digitized for each species, each taken from a different individual. In **C**, statistical difference between canopy and understory were tested using ANOVAs (**p<0.01; ***p<0.001; n.s.= not significant).

Journal of Experimental Biology • Supplementary information

Table S1. Results of phylogenetic ANOVAs testing the effect of microhabitat on climbing and wingbeat kinematic parameters. Tests performed on mean values per species (N_{canopy} = 2; $N_{understory}$=5).

Climbing kinematic parameters	Effect of microhabitat (phylogenetic ANOVA) *F*	*P*
Vertical displacement	3.21	0.133
Horizontal displacement	0.02	0.869
Vertical velocity	1.55	0.267
Horizontal velocity	0.01	0.917
Total velocity	0.12	0.734
Climb angle	1.44	0.283
Wingbeat frequency (Hz)	0.14	0.721

Table S2. Results of ANOVAs testing the effect of microhabitat and species on the wing and body kinematics parameters. ($N_{canopy\ species}$ = 2; $N_{understory\ species}$ = 2; $N_{wingbeat}$ = 3 per species, each wingbeat taken from a different individual)

Parameters	Mean value ± sd		Microhabitat		Species	
	canopy	*understory*	*F*	*P*	*F*	*P*
Wing speed (m.s.$^{-1}$)	1.27 $^{\pm 0.41}$	1.15 $^{\pm 0.53}$	0.19	0.671	1.51	0.277
Stroke amplitude (deg)	66.4 $^{\pm 1.7}$	63.9 $^{\pm 5.7}$	0.89	0.372	0.49	0.624
Forewing AoA (deg)	66.4 $^{\pm 1.7}$	63.9 $^{\pm 5.7}$	0.91	0.367	0.15	0.858
Hindwing AoA (deg)	51.6 $^{\pm 10.9}$	56.3 $^{\pm 7.6}$	1.21	0.302	3.91	0.065
Body pitch angle (deg)	66.6 $^{\pm 8.7}$	43.7 $^{\pm 9.8}$	17.88	**0.002**	0.91	0.441
